# Comparative evaluation of RNA-Seq library preparation methods for strand-specificity and low input

**DOI:** 10.1038/s41598-019-49889-1

**Published:** 2019-09-17

**Authors:** Dimitra Sarantopoulou, Soon Yew Tang, Emanuela Ricciotti, Nicholas F. Lahens, Damien Lekkas, Jonathan Schug, Xiaofeng S. Guo, Georgios K. Paschos, Garret A. FitzGerald, Allan I. Pack, Gregory R. Grant

**Affiliations:** 1 Institute for Translational Medicine and Therapeutics, University of Pennsylvania, Philadelphia, PA USA; 2 Department of Systems Pharmacology and Translational Therapeutics, University of Pennsylvania, Philadelphia, PA USA; 3 Next Generation Sequencing core, University of Pennsylvania, Philadelphia, PA USA; 4 Division of Sleep Medicine/Department of Medicine, University of Pennsylvania, Philadelphia, PA USA; 5 Department of Genetics, University of Pennsylvania, Philadelphia, PA USA

**Keywords:** Biotechnology, Computational biology and bioinformatics

## Abstract

Library preparation is a key step in sequencing. For RNA sequencing there are advantages to both strand specificity and working with minute starting material, yet until recently there was no kit available enabling both. The Illumina TruSeq stranded mRNA Sample Preparation kit (TruSeq) requires abundant starting material while the Takara Bio SMART-Seq v4 Ultra Low Input RNA kit (V4) sacrifices strand specificity. The SMARTer Stranded Total RNA-Seq Kit v2 - Pico Input Mammalian (Pico) by Takara Bio claims to overcome these limitations. Comparative evaluation of these kits is important for selecting the appropriate protocol. We compared the three kits in a realistic differential expression analysis. We prepared and sequenced samples from two experimental conditions of biological interest with each of the three kits. We report differences between the kits at the level of differential gene expression; for example, the Pico kit results in 55% fewer differentially expressed genes than TruSeq. Nevertheless, the agreement of the observed enriched pathways suggests that comparable functional results can be obtained. In summary we conclude that the Pico kit sufficiently reproduces the results of the other kits at the level of pathway analysis while providing a combination of options that is not available in the other kits.

## Introduction

RNA-Sequencing (RNA-Seq) analysis has become the *de facto* method for measuring gene expression genome wide. However, when designing an experiment, the investigator is faced with the task of making many decisions, including choice of platform and library preparation protocol, which can involve considerable trade-offs. We focus here on the Illumina and Takara Bio kits and investigate the differential effect of library prep protocol. The most widely used protocol involves the TruSeq (Illumina, catalog no. RS-122-2103) kit, which assumes abundant starting material (0.1–1 μg total RNA) and maintains strand specificity. Strand information is vital to the analysis of many studies^1–4^. Indeed many genes undergo anti-sense transcription, which serves a regulatory purpose and has also been associated with disease^5^. In a standard treatment/control experiment, an important segment of signal comes from the anti-sense strand of annotated RNA and it is not uncommon to find genes with more anti-sense signal than sense. If data generated are not strand specific, then all such reads will get quantified as “sense” signal.

In general, since one can obtain more accurate sense expression from strand specific data and one cannot obtain any anti-sense information from non-strand specific data, it is considered preferable to generate strand specific data. However, until recently it has not been possible to perform strand-specific sequencing on samples that require a high level of amplification from quantities below 10 ng total RNA. This situation has now been addressed in the new Pico kit (Takara Bio, catalog no. 634413). Different kits produce data with different biases. For example the act of removing the ribosomal RNA (rRNA) has a dramatic effect on the results; the polyA selection approach tends to result in a 3′ coverage bias, while the approach of hybridizing the rRNA out with beads results in a very different bias^6^. Therefore, the question naturally arises as to whether the same general results will be obtained from using two different kits on the same samples. To investigate this, we compared the TruSeq, Pico and V4 kits in a realistic differential expression (DE) analysis, using both abundant and minute quantities of starting material. The V4 kit (Takara Bio, catalog no. 634888) is the kit typically used for minute quantities which does not preserve strand specificity.

Most comparative analyses of RNA-Seq methods, both wet bench and computational, are on samples that are too artificial to draw meaningful conclusions about performance in practice. For example, many studies^7–10^ used the universal reference samples^11^ to benchmark differential expression (DE) analysis. Yet the comparison of technical replicates of two completely different reference samples is not realistic enough to draw practical conclusions, as we expect almost any expressed gene to be differentially expressed. Furthermore, technical replicates guarantee the distribution of each gene’s expression is independent from all other genes – which is in stark contrast to real data where a small minority of genes are differentially expressed, with an extremely complex background of non-DE genes involving population wide, highly dependent distributions.

A few recent studies assess the quality of libraries produced with low starting material using a variety of kits. Palomares *et al*. evaluate the quality of libraries produced by different input amounts, and suggest that the non-stranded V4 kit performs well for low input quantities, compared to the TruSeq kit^12^. Similarly, Song *et al*. suggest that the TruSeq and V4 kits perform well for low starting material, while they also suggest the Pico kit if polyA and non-polyA mRNA are of interest^13^. However, neither looked at the ability of the Pico kit to produce stranded data with low input material, which is its key advantage.

Here we designed a standard treatment/control experiment to assess the differences between the three kits in a typical differential expression analysis. Specifically we examined the hepatic inflammatory response of mice by assaying liver RNA from saline (control) and IL-1β treated mice^6^.

There are several levels on which the data can be compared. First is the level of the raw data itself (alignment statistics, quality scores, quantified values, ribosomal content, etc.). The second level is to compare the results of a DE analysis of genes or gene features such as exons, introns or junctions. The third level on which to compare methods is on pathway enrichment of DE genes. There might be significant discordance at the level of alignment and even the specific lists of DE genes, but if they result in the same pathway enrichment results then it is likely the same conclusions will be drawn from each analysis; and indeed, this is what was observed in this study. The greatest differences between the kits were identified at the raw data and DE levels, however for the most part no major differences were identified at the pathway level.

Although the pathway analyses were similar, one would still like to understand the source of the differences at the levels of raw data and DE. Therefore, we PCR validated some of the most discordant genes in order to determine which kit was more accurate.

## Results

Here we give the details of the comparative analysis of the three kits Pico, TruSeq and V4, on the levels of raw sequencing libraries, differential expression detection, and pathway enrichment analysis.

### Gene expression evaluation

As a standard treatment/control experiment, we sequenced three liver RNA samples from mice treated with saline and three treated with 20 μg/Kg of IL-1β. We analyzed RNA from each sample with all three kits, and used 1.7–2.6 ng of RNA with the Pico kit, 0.8–1.3 ng with the V4, and 200 ng with the TruSeq. We followed the standard steps of a typical RNA-Seq analysis. Data were aligned, normalized, quantified, and Differential Expression *p*-values were computed (see Methods). The library prep, sequencing and analysis was repeated twice for the Pico kit. Additionally, we had sequenced the same samples with the TruSeq kit for a previous study, allowing us to compare the two TruSeq runs as control for batch effects when comparing TruSeq to Pico and V4.

**Figure 1:**
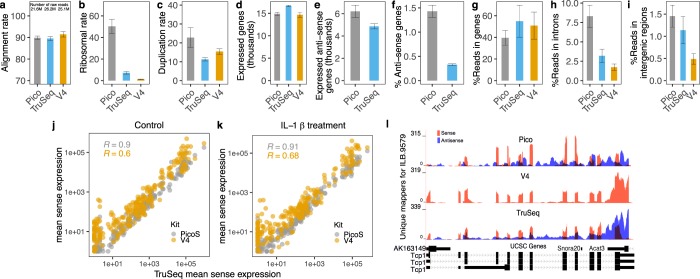
Profiling of RNA-Seq libraries. (**a**) Average rate of uniquely aligned reads to the reference genome, for each library preparation kit. The average total number of raw reads is printed for each kit in millions. (**b**) Average rate of uniquely aligned reads to ribosomal genes. TruSeq and V4 kits do polyA selection, while the Pico kit does ribo depletion using the ZapR enzyme. (**c**) Average rate of duplicated reads, after ribo removal. (**d**,**e**) Number of sense and anti-sense expressed genes per kit after normalization. We include genes that are expressed in at least one of the samples. (**f**) Percent of anti-sense reads mapping to genes out of all reads mapping to genes either sense or anti-sense, in the two stranded kits TruSeq and Pico. (**g**–**i**) Percentages of number of reads that map to gene, intron sense, and intergenic regions out of total number of reads after ribo removal. (**a**–**i**) The error bars represent the 95% confidence interval around the percentage value. The T-statistic for confidence interval for standard error was calculated. (**j**,**k**) Pairwise comparisons of mean normalized sense gene expression of the ~200 genes with the highest anti-sense expression in the TruSeq kit, in both conditions. Pearson’s correlation is calculated. This illustrates the degree to which V4 over-estimates the sense signal on such genes, because it cannot differentiate with anti-sense. (**l**) Example of *Tcp1* gene body coverage in one of the IL-1β treated samples (ILB.9579); shows that *Tcp1* has anti-sense expression (shown in blue color for Pico and TruSeq kits); indicates overestimation of the sense expression in the V4 un-stranded kit.

More than 90% of raw reads uniquely mapped to the reference genome for all three kits (Fig. 1a), which is reasonably high. Since rRNA is roughly 99% of total RNA, all kits necessarily perform ribosomal depletion. This typically removes more than 90% of rRNA, depending on tissue and other factors. Nevertheless, in the Pico kit ribosomal reads were retained up to 40–50% (Fig. 1b), while in the TruSeq kit it is ~7%. In their comparative study of ribosomal RNA removal kits, Herbert *et al*. report ~15% rRNA retention with the Pico kit^14^. Given the discrepancy we validated our findings in an independent library preparation and sequencing run. However, it is problematic to compare to Herbert *et al*., since they used very different samples - a mixture of 10 different cancer cell lines as compared to mouse liver - and used very different criteria for calling a read as ribosomal. They used a BWA alignment to the human genome build hg19 which unfortunately is missing half of 45 S, while we constructed a comprehensive rRNA library from GenBank and aligned to it using BLAST. These differences likely explain why their rRNA retention rate of 15% for the Pico kit is so much lower than ours and also why ours is probably more accurate. To investigate further, we compiled the distribution of the FPKM normalized ribosomal retention across the different ribosomal RNA subunits (5 S, 45 S, 18 S, 28 S, 5.8 S, 45 S spacers, and mitochondrial rRNAs). TruSeq and V4 have similar profiles, as expected since they both utilize polyA selection, while Pico’s ZapR method retains much higher levels of 5 S and 5.8 S (Supplemental Fig. S1).

Another common observation in RNA-Seq libraries is the high rate of duplicated reads. To evaluate this rate, we quantified the duplication rate after removal of ribosomal reads using the Duplication Rate Identifier for NGS Cleanup (https://github.com/itmat/DRINC) developed in house, and showed that it is elevated in Pico (~20%) compared to the other two kits (Fig. 1c). Overall our results indicate that the Pico kit results in substantially higher ribosomal content and PCR duplication artifacts potentially associated with the low starting material.

To assess the concordance of the kits on the level of quantification, the raw read counts were normalized and quantified at both the gene level and the exon-intron-junction levels, both sense and anti-sense (see Methods). The percentage of uniquely aligned reads that map to genes in the sense orientation (gene-mappers) in the Pico kit is ~10% smaller than in TruSeq (Fig. 1g), which can be explained by the greater intronic signal in Pico. Nonetheless, the observed number of expressed genes is comparable, across all three kits (Fig. 1d).

The ability to quantify anti-sense gene expression is the key advantage of the Pico kit over the V4 kit. To investigate the extent of anti-sense signal in the two stranded kits Pico and TruSeq, we compared the balance of sense to anti-sense gene signal and we compared how many genes were identified with anti-sense transcription. Figure 1f shows the percent of anti-sense reads mapping to genes out of all reads mapping to genes either sense or anti-sense. Surprisingly, the percent is substantially higher in Pico, with roughly 1.5% anti-sense versus roughly 0.5% in TruSeq. As a result, Pico identified about 20% more genes expressing anti-sense signal in spite of having lower read depth and considerably higher rRNA retention (Fig. 1e). We therefore conclude that Pico is at least as sensitive to anti-sense transcription as TruSeq if not more.

To investigate the adverse effect of non-strand specific sequencing on quantifying *sense* gene expression, we identified the genes with the highest anti-sense signal (~200 genes with mean anti-sense expression ≥20 normalized reads) for each treatment in the TruSeq kit. We then compared the sense expression of these genes across the three kits, to assess whether strandedness information was important for accurately quantifying gene expression levels. We expected that the correlation of the gene expression levels of TruSeq and Pico signals would be closer together because they eliminate anti-sense reads that do not constitute *bona fide* gene expression proxies. We observe that in both conditions the mean sense expression of Pico and TruSeq are highly correlated (R > 0.9), while V4 shows lower correlation with TruSeq (Fig. 1j,k); indeed, verifying that strandedness is important for accurate gene expression quantification. Figure 1l shows an example of V4 overestimating the sense gene expression; *Tcp1* gene has anti-sense expression (shown in blue for Pico and TruSeq), which is combined in the sense gene expression in the V4 results.

To investigate sense intron signal, we first identified using TruSeq which introns do not have any anti-sense signal. From these introns we compared the percentages of uniquely aligned reads between the three kits, and observed that it is elevated in the Pico kit, compared to the TruSeq and V4 kits (Fig. 1h). Similarly, we also observed an elevated percentage of reads mapping to intergenic regions in Pico, compared to the other two kits (Fig. 1i). In Supplemental Fig. S2 we illustrate these two observations for *Eri3* gene with a Genome Browser^15^ profile. This could be due to retained introns from pre-mRNA signal, since the Pico kit uses rRNA depletion instead of polyA selection.

**Figure 2:**
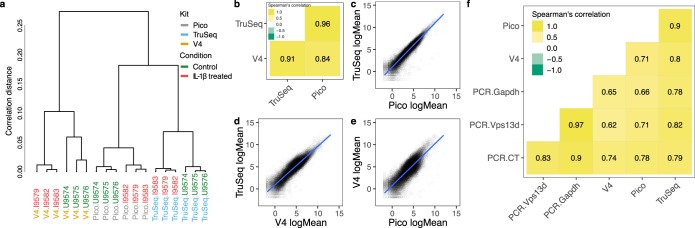
Concordance of gene expression profiles. (**a**) Hierarchical clustering by normalized expression correlation of all 18 samples shows clear distinction of the samples first by kit type and secondly by treatment. (**b**) Average sample Spearman’s correlation of normalized gene expression shows higher concordance for Pico and TruSeq. (**c**–**e**) Pairwise comparisons of mean normalized gene expression demonstrate higher concordance between Pico and TruSeq. (**f**) Spearman’s rank correlation of normalized gene expression of IL-1β treated sample (ILB.9579) indicates that TruSeq is the most highly correlated to RT-PCR, while V4 is the least. The PCR metrics shown are the number of cycles (PCR.CT), and the efficiency scores controlled by *Gapdh* (PCR.Gapdh) or *Vps13d* (PCR.Vps13d).

A hierarchical clustering of the 18 samples was performed (Fig. 2a), from which a clear distinction of kit type is observed. Thus the differences between the kits are more pronounced than the differences between the samples, in spite of a powerful treatment affecting thousands of genes with large effect sizes. After kit, samples cluster based on treatment. However, the correlation of the average of the normalized expression profiles between the three kits, as calculated using Spearman’s method, is high. Specifically, the highest correlation (0.96) is illustrated between the Pico and TruSeq kits (Fig. 2b). To further assess the concordance between the three kits, we performed pairwise comparisons of the means of normalized gene expressions (Fig. 2c–e). The higher linearity between the Pico and TruSeq kits (Fig. 2c) could be translated into the Pico kit being as reliable as the TruSeq kit.

The high concordance of the Pico and TruSeq kits was also verified by a comparison of gene rankings by average expression between the two kits (Supplemental Fig. S3; See Methods).

To further evaluate the accuracy of the kits, we validated selected genes with RT-PCR. Specifically, we used one of the IL-1β treated samples (ILB.9579), based on which we selected 18 genes (Table 1) that were highly expressed or absent in only one of the kits and not in the others. We compared the expression values of RT-PCR and RNA-Seq, using the RT-PCR efficiency score and the RNA-Seq normalized read counts of all the loci the primers mapped to, as described in Methods. Both RT-PCR and RNA-Seq data were normalized with two controls, *Gapdh* and *Vps13d* (see Methods for explanation of why *Vps13d* was used in addition to *Gapdh*). The Spearman’s rank correlation of the normalized gene expression illustrates that TruSeq is the most correlated to RT-PCR (either relative to the average number of cycles, or the efficiency score normalized by *Vps13d* or *Gapdh*), while V4 is the least (Fig. 2f).

**Table 1:** RT-PCR-validated genes.

Gene	Pico	V4	Truseq	RT-PCR (AVG CT)	Efficiency score using *Vps13d*	Efficiency score using *Gapdh*
Mup-ps19	12,839	137,475	87,486	15.68	0.007	2.037
Saa1	6,358	114,234	81,987	15.84	0.007	1.959
Gapdh	9,305	167,469	133,483	16.09	0.003	1.000
Hyou1	3,152	635	14,199	19.38	0.008	24.915
Myh9	2,893	197	3,171	20.41	0.303	90.535
Gm15450	2,035	6,377	7,295	20.72	0.234	69.974
Hist1h1e	251	58	3	20.98	0.523	127.969
Dhx9	482	57	1,217	21.25	0.245	73.192
Abhd2	808	61	2,181	21.46	0.370	110.569
Gm14681	254	594	1,417	22.35	0.294	87.770
Mll2	1,290	89	1,280	22.41	0.664	198.347
Vps13d	1,092	115	1,377	22.71	1.000	298.751
Acvr1b	160	12	488	22.72	0.453	135.227
Kif1c	581	59	1,330	23.26	1.753	523.636
Tcf20	491	16	365	23.84	5.227	1560.898
Zfp445	431	15	562	23.89	0.927	276.947
Ttll4	301	43	897	23.93	2.310	689.927
Cdc42bpb	323	11	546	25.77	0.635	189.568

The normalized gene expression of the 18 most discordant genes for sample ILB.9579 is given for all kits. The RT-PCR expression values are reported as the average number of PCR cycles, and the efficiency scores using two controls, *Gapdh* and *Vps13d*.

To summarize, in this section we have been primarily concerned with evaluating the reliability of the signal from the Pico kit once it is quantified to genes and other features such as introns and intergenic regions. The ability of Pico to measure anti-sense signal is of interest as well as the maintenance of reliable sense signal. Such measurements are what is most relevant to the downstream analysis and we conclude from the various results that the Pico kit does provide robust anti-sense signal as claimed and also that properly quantifying anti-sense signal is equally important to accurately quantifying the sense signal. In spite of the TruSeq kit having better properties at the level of raw data (e.g. alignment statistics, rRNA retention rate, etc.) it appears that the Pico kit provides sufficiently reliable sense-signal and is generally more sensitive than TruSeq to the anti-sense signal, both for gene signal and intron signal.

### Effects on differential expression identification

**Figure 3:**
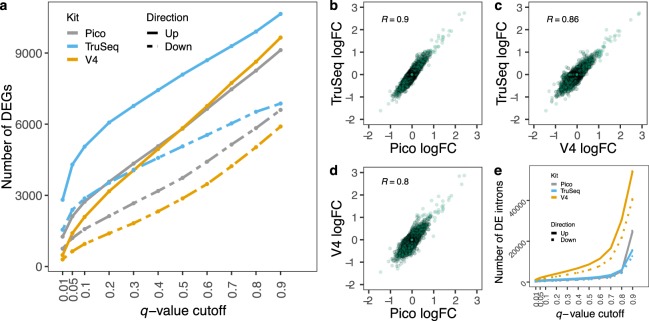
Kit effect on differential gene expression. (**a**) Number of up- and down-regulated DEGs identified at different *q*-value cutoffs. (**b**–**d**) Scatterplots of pairwise comparisons of fold changes of the average normalized gene expression, between kits. Pearson’s correlation of adjusted fold changes of the average normalized gene expression between two kits, is calculated. (**a**) Number of up- and down-regulated, differentially expressed, retained introns identified at different *q*-value cutoffs.

To evaluate the kit impact on differential gene expression, we performed differential expression analysis with *limma-voom* on both gene and intron levels, comparing untreated (controls) to IL-1β treated. For different *q*-value cutoffs, we observed that the number of differentially expressed genes (DEGs), either up-regulated or down-regulated, follow the same trend across all kits (Fig. 3a). However, it is notable that the magnitude of the number of DEGs differs significantly, with Pico identifying 55% fewer DEGs than the TruSeq kit at *q*-value cutoff 0.1, and the V4 kit even less (Fig. 3a, Table 2). In contrast, the Pico and TruSeq kits identify a small and similar number of differentially expressed, retained introns, compared to the V4 kit (Fig. 3e), which could be due to V4 incorrectly assigning anti-sense transcription to a gene’s body and introns. Moreover, the pairwise comparisons of the adjusted fold-changes (See Methods) of the average normalized gene expressions between kits also demonstrate that the Pico kit is highly concordant with the TruSeq (Fig. 3b–d).

**Table 2:** Number of up- and down-regulated, differentially expressed genes at different *q*-value cutoffs, for all kits.

*q-*value cutoff	Pico-Up	Pico-Down	V4-Up	V4-Down	Truseq-Up	Truseq-Down
0.9	9123	6600	9649	5900	10645	6869
0.8	8258	5832	8634	5028	9900	6521
0.7	7469	5140	7735	4227	9292	6031
0.6	6643	4417	6763	3479	8695	5544
0.5	5809	3737	5831	2870	8092	5064
0.4	5070	3186	4949	2325	7437	4578
0.3	4336	2667	4055	1825	6769	4075
0.2	3575	2126	3171	1397	6067	3533
0.1	2731	1578	2091	953	5053	2872
0.05	2116	1200	1404	642	4296	2390
0.01	1260	754	483	299	2815	1552

**Figure 4:**
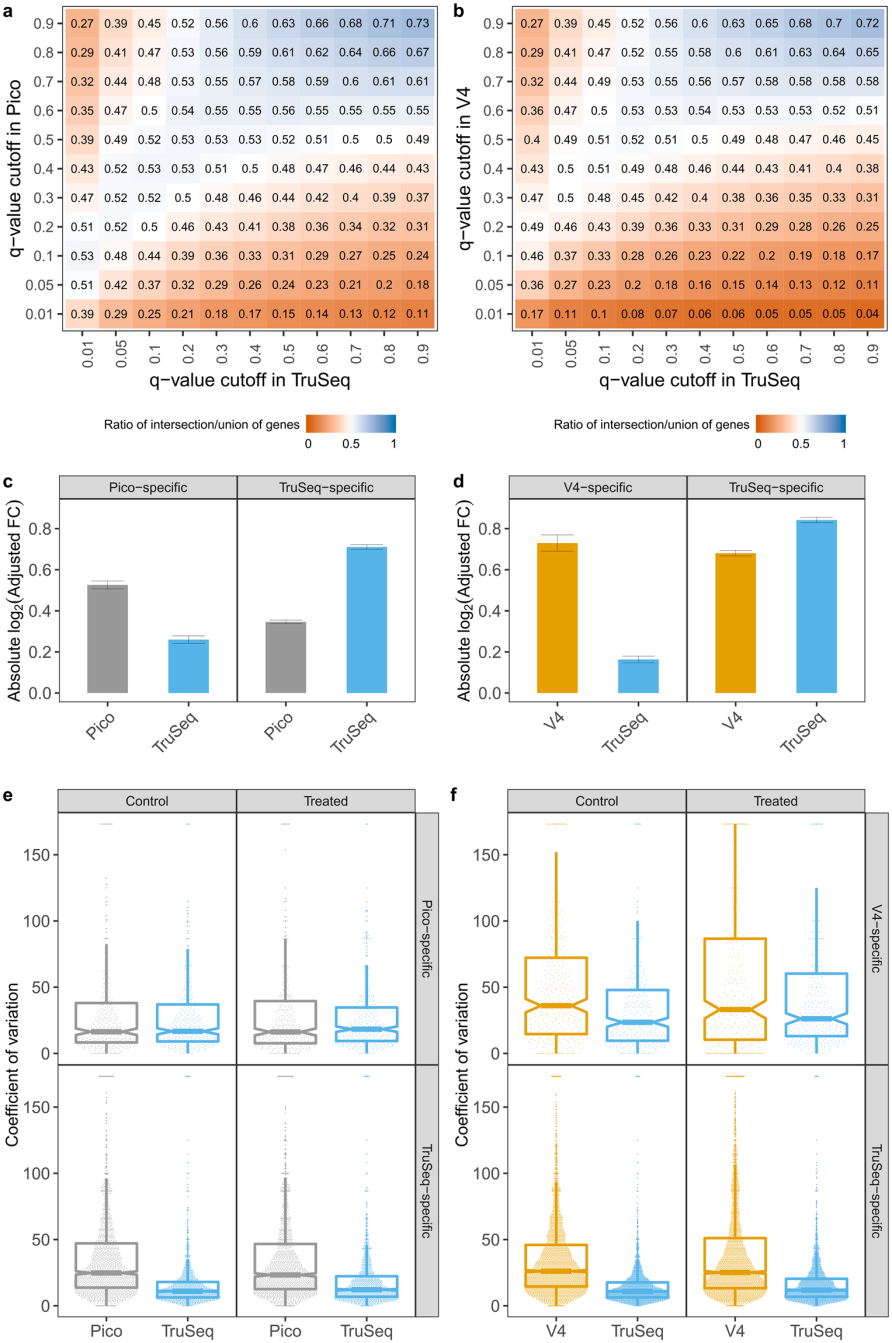
Effects on differential expression. (**a**,**b**) Heatmap of the ratio of the number of DE genes found by both kits, to the number of genes found DE by at least one of the two kits, at varying *q*-value cutoffs (blue indicates nearly identical DEG lists and orange indicates highly discordant DEG lists). (**c**) Absolute value of the log_2_ adjusted fold-changes for DEGs identified exclusively by Pico (n = 371) or TruSeq (n = 1755). (**d**) Absolute value of the log_2_ adjusted fold-changes for DEGs identified exclusively by V4 (n = 317) or TruSeq (n = 3606). (**c**,**d**) The error bars represent the 95% confidence interval around the fold-change values. DEGs identified as those with *q*-values < 0.01. (**e**) Coefficients of variation for DEGs identified exclusively by Pico or TruSeq. (**f**) Coefficients of variation for DEGs identified exclusively by V4 or TruSeq. The difference among the kits was evaluated based on the overlapping of the notch region, defined as median m ± 1.58IQR/√n (See Methods). If the notches of the boxes of the two kits do not overlap, it suggests that the medians are significantly different, with 95% confidence interval. This suggests significant differences in variation of the two kits.

We investigate why there is such a difference in the number of DEGs (Fig. 3a, Table 2), since the number of the genes being expressed is comparable across the kits (Fig. 1d). First, we examine to what extent the same DEGs are identified using the different kits. For various *q*-value cutoffs, we calculate the ratio of the number of DE genes found by both kits, to the number of genes found DE by at least one of the two kits. Values near 1 indicate that the DEG lists from both methods are nearly identical, while values near 0 indicate that only a small number of the total DEGs were found by both methods. As shown in Fig. 4a,b, we do not observe a high rate of common DEGs for any cutoffs. As a baseline for comparing V4 and Pico to TruSeq, we compared the two different runs of TruSeq (Supplemental Fig. S4a).

A decrease in power to detect DEGs is due to an increase in variance and/or a decrease in effect size (e.g. fold-change), given all other things, such as number of replicates, are equal in the study designs. We investigated the source of the differences in the DE analyses by examining those genes that were significantly DE (*q*-value ≤ 0.01) in the TruSeq data but were not significant (*q*-value > 0.01) in either the Pico or V4 kits, and conversely (Fig. 4c–f). For genes detected as significantly DE in one kit, say kit A, and not in another kit, say kit B, the fold-changes are always larger in kit A (Fig. 4c,d). Meanwhile, the coefficients of variation (CV) are always equal or lower in the TruSeq data across all comparisons (Fig. 4e,f). The V4-specfic DEGs present an interesting case, since both the CV and fold-change are substantially higher in the V4 data than in the TruSeq data. For these DEGs, it appears the increased effect size has compensated for the increased variance. However, the TruSeq data identified nearly five times more kit specific DEGs than the Pico data, and over 11 times more than the V4 data. We looked for enrichment of general properties (gene length, GC-content, number of isoforms) among the Pico- and V4-specific DEGs, compared to the TruSeq-specific DEGs, but did not identify any significant factors. We also examined effect sizes and CVs for genes not found DE (*q*-value > 0.3; mean expression per condition >2) in data from any kit (Supplemental Fig. S4b,c). Both analyses indicate that there is larger variance between the kits than between the biological replicate samples. To check for any impact of DE method on the observed DE discrepancies between kits, the DE analysis was repeated by quantifying gene expression with kallisto^16^ followed by DESeq2^17^ for the differential expression analysis. The results replicated our previous observations (Supplemental Fig. S4d).

### Implications on functional analysis

**Figure 5:**
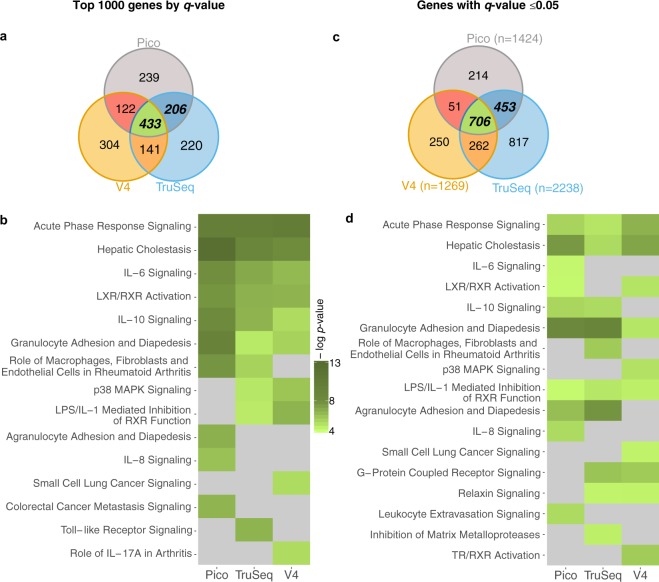
Pathway enrichment concordance. (**a**) Overlaps of the 1,000 genes, used for pathway enrichment analysis for each kit. (**b**) Heatmap of top 10 pathways of each kit, sorted by enrichment *p*-value. Pathway enrichment analysis for each kit was performed using the top 1,000 genes sorted by DE *q*-value. (**c**) Overlaps of the genes at DE *q*-value ≤ 0.05, used for pathway enrichment analysis for each kit. (**d**) Heatmap of top 10 pathways of each kit, sorted by enrichment *p*-value. Pathway enrichment analysis for each kit was performed using genes with DE *q*-value ≤ 0.05. (**b**,**d**) Grey color indicates that pathway is not in the top 10 most enriched pathways of the specific kit.

To evaluate the functional effect of the DE discrepancy, we performed pathway enrichment analysis. The pathway analysis was performed using both the top 1,000 DEGs ranked by *q*-value, and the set of genes with *q*-value ≤ 0.05. In the first analysis a modest overlap was observed (Fig. 5a). Nevertheless, all three kits led to highly similar pathway enrichments, which are primarily related to inflammatory response, as expected. The heatmap of top 10 pathways identified in each of the three kits, sorted by enrichment *p*-value is shown in Fig. 5b.

Similar results were produced using DEGs at *q*-value ≤ 0.05, for each kit (Fig. 5d), illustrating a modest overlap of genes (Fig. 5c). However, examining the two analyses (Fig. 5b,d) there is an overall consistency of the top 10 enriched pathways identified with Pico and TruSeq. Additionally, comparing the top 10 pathways identified for the Pico kit by using different *q*-value cutoffs (Fig. 5b,d), we see consistency of the enrichment analysis for Pico. V4 shows a decreased reproducibility, demonstrating a lower overlap at different *q*-value cutoff, and having a less consistent overlap when compared to TruSeq kit.

## Discussion

While RNA-seq is a powerful technology in transcriptome profiling, some protocols do not retain the strand of the original transcripts. Anti-sense transcription plays an important role in the transcriptome and additionally this strand information is critical to accurately quantify sense gene expression, particularly for genes with overlapping genomic loci that are transcribed from opposite strands^3^. With our expanding appreciation for the regulatory and biological functions provided by anti-sense transcripts, strand-specific sequencing provides the most direct means for studying this class of RNAs^4^.

Our goal was to evaluate whether the Pico kit performs as advertised. The claim is that it is comparable to the standard TruSeq kit, which is why we included the TruSeq as a gold standard. We further included the V4 just to have another baseline specific for low input materials, and also to investigate the differences of the strandedness retention. By focusing on these kits we were able to investigate the Pico kit to a level of detail which would have been cumbersome on an analysis of all kits in regular production. A broad analysis of many kits can be found in Palomares *et al*.^12^

By focusing on samples representative of what is typically sequenced in practice, and in particular representing two experimental conditions, we were able to perform a detailed comparative evaluation of three RNA-Seq library preparation kits to evaluate the effect of the strand specificity on gene expression and pathway enrichment, and therefore the efficiency of the kits that use low quantities of RNA (Pico and V4 kits) compared to the TruSeq kit.

We observe that the alignment rates are comparable with over 90% alignment rates for all kits (Fig. 1a). The Pico kit data showed elevated levels of ribosomal content (Fig. 1b), which agrees with previous findings (Takara Bio 2015, Herbert *et al*. 2018^14^), as well as increased numbers of duplicated reads (Fig. 1c) and retained introns (Fig. 1h,i). However, the gene expression profiles indicated that the Pico and TruSeq kits have the greatest concordance (Fig. 2), which was also validated with RT-PCR of the 19 most discordant genes (Fig. 2f). The number of DEGs (both up- and down-regulated) identified at varying *q*-value cutoffs, however, showed noticeable differences across kits (Fig. 3a, Table 2). Furthermore, the overlap between the sets of DEGs identified by both Pico (or V4) and TruSeq is not particularly high at any significance cutoff (Fig. 4a,b). This is likely due to the observed significant differences between the Pico (or V4) and TruSeq kits in gene expression variability across samples, for both treatment conditions (Fig. 4c–f). Specifically, Pico and V4 introduce considerably more variance than the TruSeq, which indicates that the largest variance is between the kits rather than the sample type. Finally, despite the differences observed at various levels of the comparative analysis, batch effects in preparation, and differences in sequencing geometry, the agreement of the observed enriched pathways indicates that meaningful and consistent results were obtained from using the different kits (Fig. 5b).

Additionally, we showed that the un-stranded kit overestimates the sense gene expression (Fig. 1j,k) suggesting that the best practice for accurate gene expression quantification would be to retain the strand specificity in the RNA-Seq data. We therefore recommend the Pico kit over the V4 kit for library preparation when starting with small RNA quantities. Importantly, we show that treatment-wise variation of the libraries prepared with Pico considerably affects the identification of the differentially expressed genes, suggesting that adding more replicates could result in a more powerful study design.

## Methods

### Data

Twelve-week old male C57/B6J mice were purchased from Jackson Labs and were housed in a controlled environment with regard to light, temperature and humidity in the animal facility of the University of Pennsylvania. All mice had free access to food and water. The animal care and treatment procedures were approved by the Institutional Animal Care and Use Committees of the University of Pennsylvania. All experiments were performed in accordance with relevant guidelines and regulations.

Four hours prior to tissue collection, mice were treated with either 20 µg/Kg of IL-1β or saline by intraperitoneal injection. Mice were euthanized through carbon dioxide induced asphyxiation 4 hrs after IL-1β or saline injection. Livers from animals perfused with ice-cold PBS were harvested and immediately stored in RNAlater® solution (Ambion, Austin, TX) at 4 °C. After 24 h, the tissue samples were transferred to −80 °C for storage until analysis. RNA was extracted using TRIzol® Reagent (Life Tehcnologies, Grand Island, NY) and RNeasy Kit (Qiagen, Valencia, CA) following the manufacturer’s protocol. The concentration and quality of extracted RNA were measured using NanoDrop® 1000 (Thermo Scientific, Wilmington, DE) and reverse-transcribed into cDNA using TaqMan Reverse Transcription Reagents (Applied Biosystems, Foster City, CA). Both treatment conditions have 3 biological replicates. Samples were extracted and aliquoted for the three runs, in order to assess the technical variability only.

### RNA-Sequencing

We performed RNA-Seq on 6 samples using three different library preparation kits following the manufacturer’s recommendations. UMI tags were not incorporated. (1) *Pico:* Total RNA from each liver sample was prepared for sequencing using the Takara Bio SMARTer: SMARTer® Stranded Total RNA-Seq Kit v2 - Pico Input Mammalian, with rRNA depletion, performed by first converting to cDNA using the Zapr enzyme which targets the ribosomal RNA sequences. 1.7–2.6 ng of RNA was used to prepare the RNA libraries. Five cycles of PCR were performed before rRNA depletion and fifteen cycles during the last library amplification. The libraries were sequenced on Illumina HiSeq 4000. Depths of 30–44 million paired-end 150 bp reads were generated for each sample. (2) *V4:* Total RNA from each liver sample was prepared for sequencing using the Takara Bio SMART-Seq: SMART-Seq® v4 Ultra® Low Input RNA Kit, with PolyA selection for ribo depletion. 0.8–1.3 ng of RNA was used to prepare the RNA libraries. Twelve cycles of PCR were performed during cDNA amplification and twelve cycles during Nextera library prep. The libraries were sequenced on Illumina HiSeq 4000. Depths of 15–30 million paired-end 150 bp reads were generated for each sample. (3) *TruSeq:* Total RNA from each liver sample was prepared for sequencing using the Illumina: TruSeq Stranded mRNA Sample Preparation Kit, with PolyA selection for ribo depletion. 200 ng of RNA was used to prepare the RNA libraries. Fourteen cycles of PCR were performed. The libraries were sequenced on Illumina HiSeq 2500. Depths of 16–34 million paired-end 125 bp reads were generated for each sample. The experiment with the libraries prepared with the TruSeq kit was performed by Lahens *et al*.^6^.

The Pico libraries were reproduced to repeat the experiment and ensure reproducibility.

### RT-PCR

Quantitative real time PCR was performed using Fast SYBR Green Master Mix on an ABI ViiA7 real-time PCR system in a 384 well plate at 95 °C for 20 s (hold stage), 40 cycles of 95 °C for 1 s and 60 °C for 20 s (PCR stage), and 95 °C for 15 s, 60 °C for 1 min, 95 °C for 15 s (melt curve stage). A standard curve with five different cDNA concentrations (0.125, 0.25, 0.5, 1 and 2 µl) was prepared using a representative cDNA for each gene of interest. 19 genes that were highly expressed or absent in one kit, were validated. All primers (final concentration- 1 µM/reaction) were designed based on sense signal loci using Primer3 software.

### RNA-Seq analysis

RNA-Seq data were aligned to the mouse genome build mm9 by GSNAP-v2018-07-04^18^. The raw read counts were normalized using a read-level resampling strategy, and quantified at the gene and exon-intron-junction levels, using the Pipeline Of RNA-Seq Transformations v0.8.5b-beta (PORT) (https://github.com/itmat/Normalization). We used PORT in order to obtain normalized SAM files for comparisons at the read level. For the ribosomal analysis, we aligned the reads to the ribosomal subunit sequences with Blast v2.2.30 + ^19^ and FPKM normalized the read counts. For gene quantification, reads were required to respect the annotated exon/exon junctions. This applies to both sense and anti-sense gene level quantification. Read duplication rates were calculated with Duplication Rate Identifier for NGS Cleanup (DRINC) (https://github.com/itmat/DRINC). Differential expressed genes were determined from a treated versus control comparison of first the PORT normalized expression values, using *limma-voom -v3*.*34*.*9* package^20^ and second by DESeq2 -v1.22.1^17^ applied on estimated expression by kallisto -v0.44.0^16^. Enrichment analysis was done using the Ingenuity Knowledge Base (www.ingenuity.com), on the top 1000 differentially expressed genes ranked by *q*-value. The top 10 pathways of each kit are reported. All visualization is done with R-v3.4.3 packages.

### Statistical analysis

#### Adjusted fold change

Expression fold-changes were adjusted by adding 20 reads to both terms of the ratio as suggested in Nayak *et al*.^21^ as an optimal pseudocount.

*Difference in ranks* is essentially the difference in the ranks of two kits, after sorting by the average gene expression (normalized read counts). The narrower the gene expression distributions are in Supplemental Fig. 3, the more concordant two kits are to each other.

#### Notches in boxplots

In the boxplots (Fig. 4e,f), the upper whisker extends from the hinge to the largest value no further than 1.5 * IQR from the hinge (where IQR is the inter-quartile range, or distance between the first and third quartiles). The lower whisker extends from the hinge to the smallest value at most 1.5 * IQR of the hinge.

The difference among the kits was evaluated based on the overlapping of the notch region. The notch is defined as median m ± 1.58IQR/√n^22^. This gives a roughly 95% confidence interval for comparing medians.

#### RT-PCR and RNA-Seq comparison

To compare the expression values of RT-PCR and RNA-Seq, we converted the standard curve slope given by RT-PCR to efficiency score using the following formula

$${\rm{Efficiency}}=-1+{10}^{((-1/slope))}$$

and calculated for the 19 genes, the RNA-Seq normalized read counts of all the loci the primers mapped to. We normalized both PCR and RNA-Seq data with two controls, *Gapdh* and *Vps13d*. Although *Gapdh* is a commonly used housekeeping gene, *Vps13d* appears as a cleaner control, as its primers only mapped to a single locus while the *Gapdh* primers mapped to numerous locations in several homologous genes. The efficiency scores of both controls and the number of cycles of the sample amplification are reported in Table 1. *Gm5805* was omitted as it was detected after more than 30 CT and reported high amplification value.

## Supplementary information

Supplemental_Figures

## Data Availability

All raw and processed RNA-Seq data generated in this study have been submitted to the NCBI Gene Expression Omnibus (GEO; https://www.ncbi.nlm.nih.gov/geo/) under accession number GSE124167.

## References

[1] Kim, E. J. *et al*. Complete Transcriptome Profiling of Normal and Age-Related Macular Degeneration Eye Tissues Reveals Dysregulation of Anti-Sense Transcription. *Sci*. *Rep*. **8** (2018). 10.1038/s41598-018-21104-7 PMC5813239 29445097

[2] Pelechano V, Steinmetz LM (2013). Gene regulation by antisense transcription. Nat. Rev. Genet..

[3] Zhao, S. *et al*. Comparison of stranded and non-stranded RNA-seq transcriptome profiling and investigation of gene overlap. *BMC Genomics* **16** (2015). 10.1186/s12864-015-1876-7 PMC4559181 26334759

[4] Dominic Mills J, Kawahara Y, Janitz M (2013). Strand-Specific RNA-Seq Provides Greater Resolution of Transcriptome Profiling. Curr. Genomics.

[5] Cooper TA, Wan L, Dreyfuss G (2009). RNA and Disease. Cell.

[6] Lahens NF (2017). A comparison of Illumina and Ion Torrent sequencing platforms in the context of differential gene expression. BMC Genomics.

[7] Risso D, Ngai J, Speed TP, Dudoit S (2014). Normalization of RNA-seq data using factor analysis of control genes or samples. Nat. Biotechnol..

[8] Li S (2014). Multi-platform assessment of transcriptome profiling using RNA-seq in the ABRF next-generation sequencing study. Nat. Biotechnol..

[9] Wang C (2014). The concordance between RNA-seq and microarray data depends on chemical treatment and transcript abundance. Nat. Biotechnol..

[10] Li S (2014). Detecting and correcting systematic variation in large-scale RNA sequencing data. Nat. Biotechnol..

[11] Su Z (2014). A comprehensive assessment of RNA-seq accuracy, reproducibility and information content by the Sequencing Quality Control Consortium. Nat. Biotechnol..

[12] Palomares, M. A. *et al*. Systematic analysis of TruSeq, SMARTer and SMARTer Ultra-Low RNA-seq kits for standard, low and ultra-low quantity samples. *Sci*. *Rep*. **9** (2019). 10.1038/s41598-019-43983-0 PMC6525156 31101892

[13] Song, Y. *et al*. A comparative analysis of library prep approaches for sequencing low input translatome samples. *BMC Genomics* **19** (2018). 10.1186/s12864-018-5066-2 PMC6151020 30241496

[14] Herbert, Z. T. *et al*. Cross-site comparison of ribosomal depletion kits for Illumina RNAseq library construction. *BMC Genomics* **19** (2018). 10.1186/s12864-018-4585-1 PMC6389247 29703133

[15] Kent W (2002). UCSC Genome Browser. Hum. genome Brows. UCSC. Genome Res..

[16] Bray NL, Pimentel H, Melsted P, Pachter L (2016). Near-optimal probabilistic RNA-seq quantification. Nat. Biotechnol..

[17] Love, M. I., Huber, W. & Anders, S. Moderated estimation of fold change and dispersion for RNA-seq data with DESeq. 2. *Genome Biol*. **15** (2014). 10.1186/s13059-014-0550-8 PMC4302049 25516281

[18] Wu TD, Reeder J, Lawrence M, Becker G, Brauer MJ (2016). GMAP and GSNAP for genomic sequence alignment: Enhancements to speed, accuracy, and functionality. in. Methods in Molecular Biology.

[19] Altschul SF, Gish W, Miller W, Myers EW, Lipman DJ (1990). Basic local alignment search tool. J. Mol. Biol..

[20] Ritchie ME (2015). Limma powers differential expression analyses for RNA-sequencing and microarray studies. Nucleic Acids Res..

[21] Nayak, S. *et al*. Iso-relevance Functions - A Systematic Approach to Ranking Genomic Features by Differential Effect Size. *bioRxiv* 381814, 10.1101/381814 (2018).

[22] McGill R, Tukey JW, Larsen WA (1978). Variations of box plots. Am. Stat..

