# Supplementary material for: Comparative evaluation of RNA-Seq library preparation methods for strand-specificity and low input
Source: Sci Rep. 2019 Sep 17;9:13477. doi: 10.1038/s41598-019-49889-1 (PMC6748930; doi:10.1038/s41598-019-49889-1)
Supplement: Supplementary file 1 — Supplemental_Figures [file 41598_2019_49889_MOESM1_ESM.pdf]

Comparative evaluation of RNA-Seq library preparation methods for strand-specificity and low input

Dimitra Sarantopoulou[1], Soon Yew Tang[2], Emanuela Ricciotti[2], Nicholas F. Lahens[1], Damien Lekkas[2], Jonathan Schug[3], Xiaofeng S. Guo[4], Georgios K. Paschos[2], Garret A. FitzGerald[1,2], Allan I. Pack[4], Gregory R. Grant[1,5,*]

1. Institute for Translational Medicine and Therapeutics 2. Department of Systems Pharmacology and Translational Therapeutics 3. Next Generation Sequencing core 4. Center for Sleep and Circadian Neurobiology 5. Department of Genetics; All affiliations at University of Pennsylvania, Philadelphia, PA, USA

Supplemental Material – Table of contents

Supplemental Fig. S1 – Distribution of the retained ribosomal signal 2
Supplemental Fig. S2 - Retained intron signal in Pico 3
Supplemental Fig. S3 - Concordance of gene expression profiles 4
Supplemental Fig. S4 - Effects on differential expression 5

Supplemental Fig. S1 – Distribution of the retained ribosomal signal

The distribution of the FPKM normalized ribosomal retention among the different ribosomal RNA subunits (5S, 45S, 18S, 28S, 5.8S, 45S spacers, and mitochondrial rRNAs).

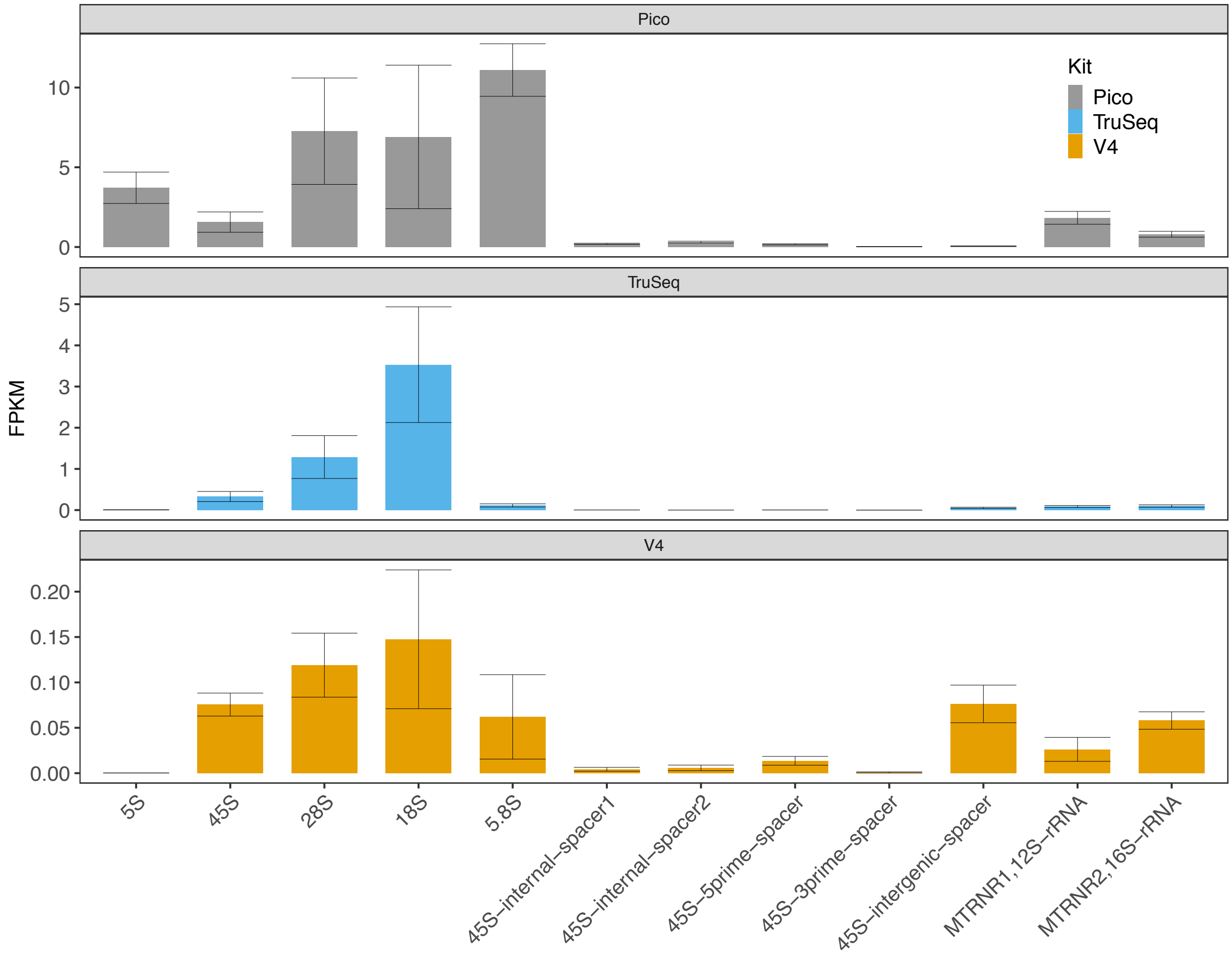

Supplemental Fig. S2 - Retained intron signal in Pico

Example of *Eri3* gene body coverage in one of the IL-1β treated samples (ILB.9579) illustrates high intron signal retained in Pico.

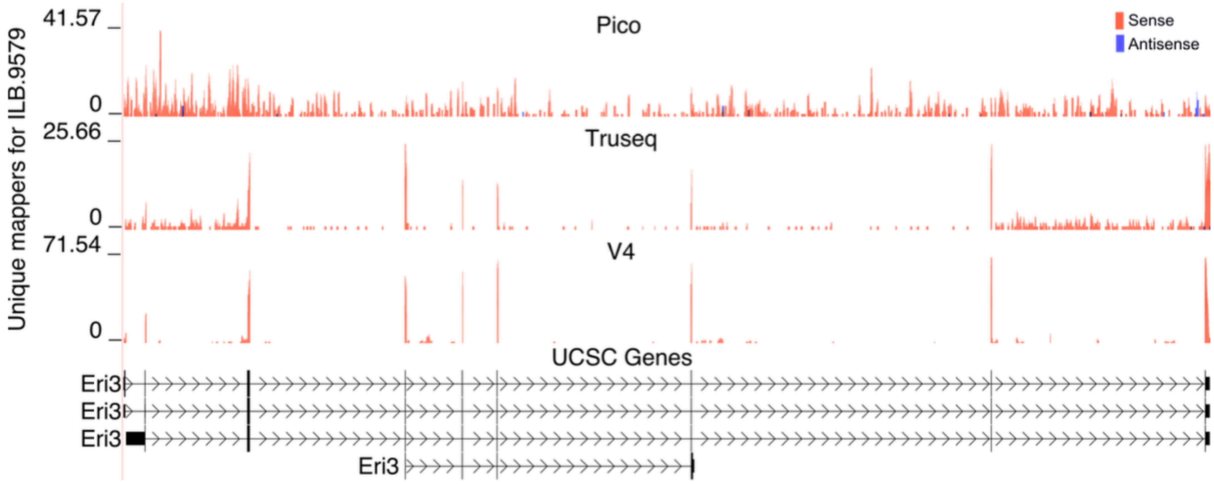

Supplemental Fig. S3 - Concordance of gene expression profiles

The concordance of gene expression distribution between two kits is shown by the difference in ranks of two kits, sorting by average gene expression. The narrower the gene expression distributions are, the more concordant two kits are to each other. The X-axis represents the value of the difference in row ranks in linear scale.

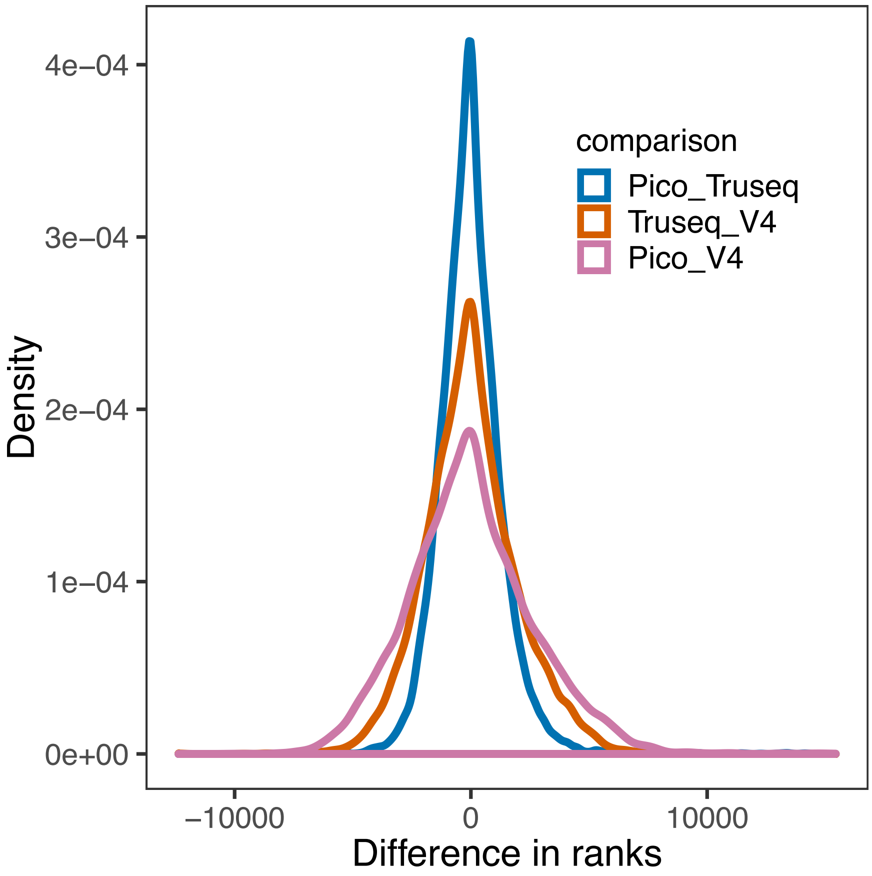

Supplemental Fig. S4 - Effects on differential expression

a) As a control, a second round of TruSeq library prep, sequencing, and analysis were performed on the same samples. This heatmap displays the ratio of the number of DE genes found by both TruSeq datasets, to the number of genes found DE in at least one, at varying q-value cutoffs. We observe high overlap of DEGs at all significant cutoffs, which suggests that TruSeq identifies a similar set of DEGs in different sequencing runs. b) Absolute value of the log2 adjusted fold-change values for the set of non-DEGs at q-value > 0.3. c) Coefficient of variation within each condition for the set of non-DEGs at q-value > 0.3 and mean expression per condition > 2. The CVs of both conditions are summarized per kit. b-c) The error bars represent the 95% confidence intervals around the mean fold-change and CV values. d) Heatmap of the ratio of the number of DE genes found by both kits, to the number of genes found DE by at least one of the two kits, at varying q-value cutoffs, for the repeated DE analysis with DESeq2.

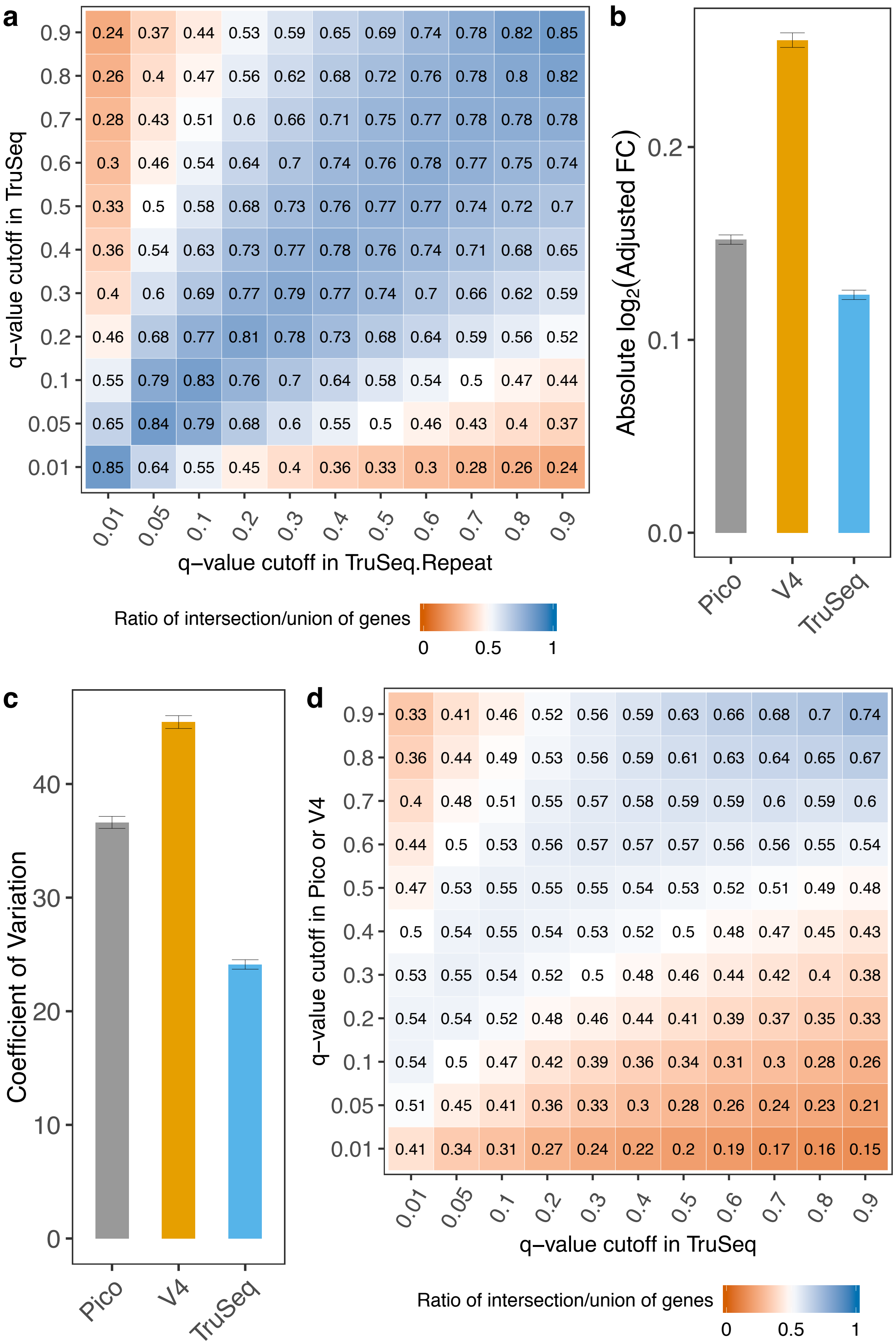
a
q−value cutoff in TruSeq
0.9 0.24 0.37 0.44 0.53 0.59 0.65 0.69 0.74 0.78 0.82 0.85
0.8 0.26 0.4 0.47 0.56 0.62 0.68 0.72 0.76 0.78 0.8 0.82
0.7 0.28 0.43 0.51 0.6 0.66 0.71 0.75 0.77 0.78 0.78 0.78
0.6 0.3 0.46 0.54 0.64 0.7 0.74 0.76 0.78 0.77 0.75 0.74
0.5 0.33 0.5 0.58 0.68 0.73 0.76 0.77 0.77 0.74 0.72 0.7
0.4 0.36 0.54 0.63 0.73 0.77 0.78 0.76 0.74 0.71 0.68 0.65
0.3 0.4 0.6 0.69 0.77 0.79 0.77 0.74 0.7 0.66 0.62 0.59
0.2 0.46 0.68 0.77 0.81 0.78 0.73 0.68 0.64 0.59 0.56 0.52
0.1 0.55 0.79 0.83 0.76 0.7 0.64 0.58 0.54 0.5 0.47 0.44
0.05 0.65 0.84 0.79 0.68 0.6 0.55 0.5 0.46 0.43 0.4 0.37
0.01 0.85 0.64 0.55 0.45 0.4 0.36 0.33 0.3 0.28 0.26 0.24
0.01 0.05 0.1 0.2 0.3 0.4 0.5 0.6 0.7 0.8 0.9
q−value cutoff in TruSeq.Repeat
Ratio of intersection/union of genes
0 0.5 1
b
Absolute $\log_2$(Adjusted FC)
0.0 0.1 0.2
Pico V4 TruSeq
c
Coefficient of Variation
0 10 20 30 40
Pico V4 TruSeq
d
q−value cutoff in Pico or V4
0.9 0.33 0.41 0.46 0.52 0.56 0.59 0.63 0.66 0.68 0.7 0.74
0.8 0.36 0.44 0.49 0.53 0.56 0.59 0.61 0.63 0.64 0.65 0.67
0.7 0.4 0.48 0.51 0.55 0.57 0.58 0.59 0.59 0.6 0.59 0.6
0.6 0.44 0.5 0.53 0.56 0.57 0.57 0.57 0.56 0.56 0.55 0.54
0.5 0.47 0.53 0.55 0.55 0.55 0.54 0.53 0.52 0.51 0.49 0.48
0.4 0.5 0.54 0.55 0.54 0.53 0.52 0.5 0.48 0.47 0.45 0.43
0.3 0.53 0.55 0.54 0.52 0.5 0.48 0.46 0.44 0.42 0.4 0.38
0.2 0.54 0.54 0.52 0.48 0.46 0.44 0.41 0.39 0.37 0.35 0.33
0.1 0.54 0.5 0.47 0.42 0.39 0.36 0.34 0.31 0.3 0.28 0.26
0.05 0.51 0.45 0.41 0.36 0.33 0.3 0.28 0.26 0.24 0.23 0.21
0.01 0.41 0.34 0.31 0.27 0.24 0.22 0.2 0.19 0.17 0.16 0.15
0.01 0.05 0.1 0.2 0.3 0.4 0.5 0.6 0.7 0.8 0.9
q−value cutoff in TruSeq
Ratio of intersection/union of genes
0 0.5 1
